# The household contact study design for genetic epidemiological studies of infectious diseases

**DOI:** 10.3389/fgene.2013.00061

**Published:** 2013-04-30

**Authors:** Catherine M. Stein, Noémi B. Hall, LaShaunda L. Malone, Ezekiel Mupere

**Affiliations:** ^1^Department of Epidemiology and Biostatistics, Case Western Reserve UniversityCleveland, OH, USA; ^2^Uganda – Case Western Reserve University Research CollaborationKampala, Uganda; ^3^Mulago Hospital, Makerere University School of MedicineKampala, Uganda

**Keywords:** extended pedigrees, genetic association, whole genome studies, cohort study, case-contact

## Abstract

Most genetic epidemiological study designs fall into one of two categories: family based and population-based (case–control). However, recent advances in statistical genetics call for study designs that combine these two approaches. We describe the household contact study design as we have applied it in our several years of study of the epidemiology of tuberculosis. Though we highlight its applicability for genetic epidemiological studies of infectious diseases, there are many facets of this design that are appealing for modern genetic studies, including the simultaneous enrollment of related and unrelated individuals, closely and distantly related individuals, collection of extensive epidemiologic and phenotypic data, and evaluation of effects of shared environment and gene by environment interaction. These study design characteristics are particularly appealing for current sequencing studies.

## INTRODUCTION

The advantages of family studies for genetic epidemiology have long been established (Stein and Elston, 2009). Early methods in genetic epidemiology utilized twins, sibling pairs, and other relative pairs to establish the relative recurrence risk of a disease. Segregation analysis and traditional linkage analysis can only be conducted using pedigree data. Concerns of population stratification are easily accounted for. In addition to these analytical issues, family studies have the advantage of investment of relatives; if someone in the family has a particular disease, family members are more likely to participate in research in order to somehow help their relative and others affected with the disease. Today, with the advent of whole exome and whole genome sequencing technologies, there are additional advantages of family studies, which we shall review below.

These advantages of family studies are further amplified for genetic epidemiological studies of infectious diseases. It was once believed that tuberculosis (TB) was a familial disease because it occurred within families. Once the disease was determined to be caused by a mycobacteria, the ideas surrounding the familial component recessed to the background. Now decades after the causal pathogen, *Mycobacterium tuberculosis* (Mtb), has been identified, many studies have shown that human genetic factors influence risk for development of TB infection and disease (Moller and Hoal, 2010; Stein, 2011). Development of TB infection and disease is essentially a phenotype resulting from a gene by environment interaction, so a well-constructed genetic epidemiological study must account for host genetics, shared environment, and gene x environment interaction. In this paper, we provide an overview of our household contact (HHC) study of TB and its advantages for genetic epidemiological studies, particularly in light of study designs best suited to identify rare genetic variants.

## OVERVIEW OF THE HOUSEHOLD CONTACT STUDY DESIGN

In its natural history, TB is a two-stage process of infection followed by disease (Comstock, 1982). The household provides a natural setting to study TB because the genetic epidemiology of the two stages of infection and disease can be characterized. In our previous studies (Guwatudde et al., 2003), we defined a household as a group of people living within one residence and share meals together with a head of family who makes decisions for the household. Extensive epidemiological data are collected on individual risk factors, such as proximity and frequency of contact with the index case as well as other factors that may increase susceptibility, characteristics of the home that may increase the risk of transmission, as well as clinical data. Blood samples are obtained at baseline and longitudinally for genetic and immunologic studies.

In our HHC study, the first TB patient is identified in the household and referred to as the index case. Thereafter individuals who reside in the same household with the index case for a certain period prior to the diagnosis of the index case are identified and screened for TB as HHCs. Each HHC is also evaluated clinically for latent Mtb infection with the tuberculin skin test (or interferon-γ response assay in the future). Individuals who are tuberculin skin test negative have repeated skin tests several times over the 2-year study follow-up. Thus, the HHC evaluation is efficient in identification of individuals with different phenotypes or stages of TB infection in a household including: (1) exposed and uninfected, (2) exposed and infected without disease, (3) recent infection, and (4) active TB. These different household phenotypes or categories can provide the basis to compare genetic factors associated with TB infection and disease. As all of these stages of infection and disease are diagnosed, both the index case and his/her contacts receive appropriate clinical care and treatment, which is an immediate benefit to all study participants.

The design of the HHC study is ideal for evaluating genetic susceptibility to TB (Stein et al., 2003, 2005, 2007, 2008). The family structure and the ability to identify sibling pairs can form the basis for linkage analysis studies. Evaluation for new candidate genes for TB can be done through conduct of association studies such as case–control, family based, and/or case–parent studies. Heritability to TB can be determined using standard quantitative genetic approaches which can be based on host immune responses as intermediate phenotypes (Stein et al., 2005; Tao et al., 2013). Studies of HHCs have demonstrated that young children are at greater risk for developing TB and the clustering of cases within families does give hint at a familial susceptibility (Brailey, 1940; Puffer et al., 1952).

In sum, the essence of an HHC design is the recruitment of an entire household through an index case/proband, and collection of extensive clinical and epidemiological data. All age ranges and relative pair types are enrolled, and the entire spectrum of disease is captured. There is flexibility for collection of biological samples and a longitudinal component to observe changes in phenotypes and biomarkers.

## ADVANTAGES OF THE HHC DESIGN FOR CURRENT GENETIC EPIDEMIOLOGICAL STUDIES

### RECRUITMENT AND PHENOTYPE COLLECTION

As summarized above, the household is ascertained through an index case with TB (aka proband). Thus, as long as each individual in the household provides informed consent (or assent in the case of children), an entire family is enrolled in the study. Sometimes, there is another individual with TB in the household at the time of enrollment (co-prevalent case). In some households, another individual develops TB later on during the course of study follow-up (incident case). In this respect, no additional recruitment efforts are needed to identify additional affected individuals. The longitudinal component of the HHC design is valuable, especially for TB, where individuals have a 5–10% lifetime risk of developing active disease after exposure. In our studies, we have observed incident cases develop 2 years after initial enrollment of the household. If related individuals are desired for analytical and study design reasons (see “Analytical Considerations” below), the HHC design allows for easier enrollment of relatives, particularly in settings where literacy is low and roads are impassable (Bennett et al., 2002). Since both HIV co-infected and uninfected individuals may live within the same household, both will be enrolled in the study; this enables the examination of gene by HIV interaction effects (Stein et al., 2007). Finally, the ideal setting for a case-contact study is where the balance of household vs. community spread of disease is in favor of the household (Hill and Ota, 2010).

Both pediatric and adult TB cases may be diagnosed because the HHC design does not restrict enrollment by age. Studies suggest that the genetic influences on pediatric vs. adult TB differ (Malik et al., 2005; Alcais et al., 2010) and the HHC study design is an efficient method for ascertaining both types of cases. By contrast, studies that focus solely on recruitment of pediatric TB cases are challenging – school-based studies are limited because children living in poverty may not have access to education, and hospital- and clinic-based studies may also miss out on enrolling children because many babies are born at home in developing countries and families in poverty who are most at risk for developing TB may not have access to medical care. Door-to-door case finding strategies would require a great number of resources in order to identify a sufficient number of pediatric cases.

The HHC design also enables the enrollment of appropriate “controls.” For a proper case–control study, controls must be similar in every way to the cases except that they do not have the disease of interest. For infectious diseases like TB, this is especially true, and in order for an individual to have the opportunity to become a case, he/she must have been exposed to an infectious TB case. This is particularly important for TB, because clinical status of the controls determines whether observed genetic associations are with susceptibility to latent infection or progression to active disease (Stein, 2011). By virtue of the HHC design, all the household members have been exposed to the index case. The selection of appropriate controls in community-based studies of TB is problematic (Hill and Ota, 2010).

Finally, studies of large pedigrees often include extensive and highly detailed phenotype information (Wijsman, 2012). This is extraordinarily useful for infectious diseases such as TB for a number of reasons. As the natural history of Mtb infection and disease follows a two-stage process, the longitudinal HHC design captures all of these stages, and progression from one stage to another. Furthermore, the HHC design can also include collection of extensive immunological data. The HHC design therefore is flexible enough to analyze immunological correlates of the natural history of TB (Whalen et al., 2006; Mahan et al., 2012), and also genetic influences on the immune response to Mtb (Stein et al., 2007, 2008). Omics technologies, such as gene expression and proteomic arrays, can also be incorporated into a study that has an established blood draw protocol and rigorous clinical classification. Finally, as we describe later, data are also collected on important epidemiological factors, which can be incorporated as covariates as well as in gene by environment interaction models.

### ANALYTICAL CONSIDERATIONS

One unique aspect of HHC studies is that households may contain all sorts of relationship types – nuclear families, extended relatives, and unrelated individuals. Half-siblings are common in African settings where polygamy is practiced (Bennett et al., 2002). Similarly, adoption by extended relatives is common when children are orphaned, which may be particularly relevant in areas with a heavy AIDS burden.

A few studies have developed strategies for jointly analyzing family based and case–control/population-based data (Chen and Lin, 2008; Gray-McGuire et al., 2009; Lasky-Su et al., 2010; Zheng et al., 2010; Mirea et al., 2012). Though they differ in how they combine data from these two different study designs – some analyze them all together, and some combine *p*-values or test statistics – there are some common themes. First, joint analysis of data from these two different study designs results in increased power due to increased sample size, enabling the detection of smaller effect sizes. Second, family based data have the advantage of controlling for population substructure, which alleviates this common concern of population-based studies.

There have been many recent reports detailing the usefulness of extended pedigrees for the analysis of sequence data and detection of rare variants. Cirulli and Goldstein (2010) explain how the analysis of distantly related, co-affected individuals is an economical design, because there will be fewer genetic variants in common, thereby reducing the search space for rare variants. Stringent filtering could use identity-by-descent sharing to capitalize on this biological phenomenon (Akula et al., 2011). Large pedigrees also have increased power to detect linkage, even in the presence of linkage heterogeneity among families, and are enriched for variants of interest (Wijsman, 2012). Linkage analysis with pedigree data can be used as a filtering strategy of chromosomal regions, and can guide the selection of subjects to sequence (Wijsman, 2012). In addition, linkage analysis may be conducted to examine co-segregation between the trait and variant(s) of interest (Clerget-Darpoux and Elston, 2007; Ziegler and Sun, 2012). Consanguineous marriages are common in West Africa, which increases the power to detect rare recessive alleles (Bennett et al., 2002). To summarize, all of the relationship types that are useful for the identification of rare variants are easily obtainable in the HHC design.

### IMPACT OF ENVIRONMENT

A well-designed HHC study includes vast epidemiologic data about environmental risk factors for transmission of disease within homes. For TB, these include factors related to ventilation and crowding within the home, poverty, clinical characteristics of the index case that make him/her more infectious, and proximity to the index case that increase degree of contact (Stein et al., 2005; Mandalakas et al., 2012). Risk of infection by Mtb is determined by a number of epidemiological risk factors (Guwattude et al., 2003; Lienhardt et al., 2003; Mandalakas et al., 2012), and many variables associated with high risk of TB transmission are automatically present in the HHC design. Analysis of foster relationships as seen in adoptions may be useful for the estimation of effects due to shared environment (Bennett et al., 2002), and many such relationships occur in HHC studies in the developing world.

Genetic substrains of Mtb may differ in their transmissibility. All of these factors relate to the risk of an individual to acquire infection, and develop disease, and thus are important in epidemiological characterization of affected individuals. Furthermore, recent studies have also suggested that substrains of Mtb have synergistic effects with host genes, thus resulting in gene x environment interaction effects related to TB risk (Caws et al., 2008). Case-only designs can be nested within HHC studies to examine these gene x environment effects (Bennett et al., 2002). Because exposure to the index case is generally highest, and in turn exposure to that individual’s strain of Mtb, the HHC design provides a natural setting to test both transmissibility, gene x environment interaction, and role of shared environment.

Nutrition and nutritional status are also important factors in TB-related outcomes (Jaganath and Mupere, 2012; Mupere et al., 2012a). We have shown that nutritional status of a patient may be an indicator on how the food basket is shared in the household and the subsequent macro- and micronutrient intake (Mupere et al., 2012b). Because of the shared environmental and genetic components of diet and obesity (or in the case of TB, malnutrition), the HHC design provides a robust setting to test the role of nutritional status on infectious disease outcomes.

### EXAMPLES FROM OUR STUDIES

Our genetic association studies have taken the approach by Gray-McGuire et al. (2009). We identified the first reported association between TNFR1 gene and TB and also a gene by HIV interaction for this same gene (Stein et al., 2007). Our genome-wide linkage scan (Stein et al., 2008) and subsequent fine mapping studies (Baker et al., 2011) replicated previously a novel set of genes on chromosome 20, CTSZ, and MC3R. We have also identified novel chromosomal regions linked to a unique resistance phenotype (Stein et al., 2008); we are uniquely able to clinically and epidemiologically characterize this phenotype because of our solid study design. Our future plans will incorporate structural equation modeling (SEM to multivariately analyze the influences of host genetics, immunology, and environment on clinical outcome; this shall be done using a SEM approach that jointly models familial relationship and covariance among variables (Morris et al., 2011).

## CONCLUSION

Certainly HHC designs may be expensive to implement, because they include repeated clinical visits, longitudinal data collection, and travel to the homes. However, the wealth of data collected through HHC studies is invaluable for genetic epidemiological studies, as described here. HHC study designs offer unique advantages for genetic epidemiological studies, including the presence of related and unrelated individuals, and the ability to quantify environmental factors that are important for both shared environmental influences on the phenotype as well as gene x environment interaction. Though our focus has been primarily on studies of TB, this study design has advantages for the study of infectious diseases in general (Hill and Ota, 2010).

## Conflict of Interest Statement

The authors declare that the research was conducted in the absence of any commercial or financial relationships that could be construed as a potential conflict of interest.
